# Proteomic analyses of smear-positive/negative tuberculosis patients uncover differential antigen-presenting cell activation and lipid metabolism

**DOI:** 10.3389/fcimb.2023.1240516

**Published:** 2023-10-16

**Authors:** Yingjiao Ju, Chengji Jin, Shan Chen, Jie Wang, Cuidan Li, Xiaotong Wang, Peihan Wang, Liya Yue, Xiaoyuan Jiang, Bahetibieke Tuohetaerbaike, Ying Li, Yongjie Sheng, Wushou’er Qimanguli, Jing Wang, Fei Chen

**Affiliations:** ^1^ Chinese Academy of Sciences (CAS) Key Laboratory of Genome Sciences and Information, Beijing Institute of Genomics, Chinese Academy of Sciences and China National Center for Bioinformation, Beijing, China; ^2^ College of Life Sciences, University of Chinese Academy of Sciences, Beijing, China; ^3^ Department of Respiratory Medicine, Second Affiliated Hospital of Hainan Medical University, Haikou, Hainan, China; ^4^ Respiratory Department, First Affiliated Hospital of Xinjiang Medical University, State Key Laboratory of Pathogenesis, Prevention and Treatment of High Incidence Diseases in Central Asia, Urumqi, Xinjiang, China; ^5^ Key Laboratory for Molecular Enzymology and Engineering of Ministry of Education, School of Life Sciences, Jilin University, Changchun, China; ^6^ Department of Respiratory Medicine, Second Affiliated Hospital of Xinjiang Medical University, Urumqi, Xinjiang, China; ^7^ Beijing Key Laboratory of Genome and Precision Medicine Technologies, Beijing Institute of Genomics, Chinese Academy of Sciences and China National Center for Bioinformation, Beijing, China

**Keywords:** tuberculosis, smear-negative pulmonary tuberculosis, smear-positive pulmonary tuberculosis, proteomics, antigen-presenting cells

## Abstract

**Background:**

Tuberculosis (TB) remains a major global health concern, ranking as the second most lethal infectious disease following COVID-19. Smear-Negative Pulmonary Tuberculosis (SNPT) and Smear-Positive Pulmonary Tuberculosis (SPPT) are two common types of pulmonary tuberculosis characterized by distinct bacterial loads. To date, the precise molecular mechanisms underlying the differences between SNPT and SPPT patients remain unclear. In this study, we aimed to utilize proteomics analysis for identifying specific protein signatures in the plasma of SPPT and SNPT patients and further elucidate the molecular mechanisms contributing to different disease pathogenesis.

**Methods:**

Plasma samples from 27 SPPT, 37 SNPT patients and 36 controls were collected and subjected to TMT-labeled quantitative proteomic analyses and targeted GC-MS-based lipidomic analysis. Ingenuity Pathway Analysis (IPA) was then performed to uncover enriched pathways and functionals of differentially expressed proteins.

**Results:**

Proteomic analysis uncovered differential protein expression profiles among the SPPT, SNPT, and Ctrl groups, demonstrating dysfunctional immune response and metabolism in both SPPT and SNPT patients. Both groups exhibited activated innate immune responses and inhibited fatty acid metabolism, but SPPT patients displayed stronger innate immune activation and lipid metabolic inhibition compared to SNPT patients. Notably, our analysis uncovered activated antigen-presenting cells (APCs) in SNPT patients but inhibited APCs in SPPT patients, suggesting their critical role in determining different bacterial loads/phenotypes in SNPT and SPPT. Furthermore, some specific proteins were detected to be involved in the APC activation/acquired immune response, providing some promising therapeutic targets for TB.

**Conclusion:**

Our study provides valuable insights into the differential molecular mechanisms underlying SNPT and SPPT, reveals the critical role of antigen-presenting cell activation in SNPT for effectively clearing the majority of *Mtb* in bodies, and shows the possibility of APC activation as a novel TB treatment strategy.

## Introduction

Tuberculosis (TB) is a significant global health concern and the second most deadly infectious disease after COVID-19, ranking as the 13th leading cause of death worldwide [Huang et al., 2019; World Health Organization, 2022]. In 2022, approximately 10.6 million individuals suffered from TB worldwide, and the disease claimed the lives of 1.6 million individuals [World Health Organization, 2022]. Smear-negative pulmonary tuberculosis (SNPT) and smear-positive pulmonary tuberculosis (SPPT) are two common types of active tuberculosis (Behr et al., 1999; Sossen et al., 2023).They are distinguished through microscopic examination outcomes of sputum samples for acid-fast bacilli (Dutt and Stead, 1994; Sossen et al., 2023). Generally, SPPT and SNPT correspond to relative high and low bacterial loads within the body, respectively (Dutt and Stead, 1994), with SPPT manifesting more severe symptoms and higher infectiousness than SNPT (Murthy et al., 2018; Kendall et al., 2021).

SPPT and SNPT can interconvert along with bacterial load changes. Some SNPT patients may progress to SPPT when the bacterial load increases to a detectable level in sputum smear microscopy (Colebunders and Bastian, 2000; Sossen et al., 2023). Conversely, SPPT can also convert to SNPT after treatment (Xu et al., 2016; Asemahagn, 2021), serving as an essential interim indicator of the effectiveness of the anti-TB treatment regimen and the reduction of infectivity (Calderwood et al., 2021; Yang et al., 2022). This conversion process can be influenced by various factors such as disease severity, host’s immune status, and drug treatment effectiveness (Wang et al., 2009; Lee et al., 2012).

Previous studies have also reported some molecular mechanisms of SPPT and SNPT (Mwandumba et al., 2008; Chen et al., 2023; Yang and Feng, 2023). Yang et al. found that SNPT patients exhibited fewer pulmonary cavities and milder inflammatory responses, with lower numbers of immune cells and higher numbers of B-cells (Yang and Feng, 2023). Mwandumba et al. observed higher pro-inflammatory and immunomodulatory cytokine responses in SNPT than in SPPT, based on cellular and cytokine profile analyses in bronchoalveolar lavage (BAL) fluids (Mwandumba et al., 2008). Additionally, Chen et al. unveiled a higher degree of cellular exhaustion in SNPT compared to SPPT by flow cytometry in peripheral blood and bronchoalveolar lavage fluid of SNPT and SPPT patients (Chen et al., 2023).

Overall, although previous studies about SPPT and SNPT reveal some differential molecular mechanisms between the two clinical phenotypes, a comprehensive analysis of the panoramic molecular mechanisms remains absent. An in-depth comprehension of differential molecular mechanisms underlying SNPT and SPPT can provide valuable insights into disease progression, pathogenesis and new treatment strategies for TB.

Proteomics provides a powerful tool for analyzing molecular mechanisms of various disease processes at the protein level(Patterson and Aebersold, 2003). Numerous have reported on molecular mechanism analyses using the sera or plasma proteomic data from active TB patients. Li et al. illuminated the altered apoptosis, blood coagulation, and oxidative phosphorylation in *Mtb* infected macrophages through quantitative proteomics (Li et al., 2017); Arya R et al. demonstrated altered immune responses and lipid metabolism within small extracellular vesicles derived from TB patient serum (Arya et al., 2020); Similarly, Mateos J et al. delineated the up-regulation of complement and immune responses, alongside the down-regulation of lipid transport and iron assimilation in active TB patients (Mateos et al., 2020). Moreover, several studies have focused on the discovery of proteomic biomarkers for TB diagnosis. For instance, Achkar JM et al. identified a set of ten candidates that could distinguish TB from non-TB respiratory diseases with high accuracy (Achkar et al., 2015); Dao TL et al. demonstrated the precise differentiation between TB and non-TB respiratory infections through MALDI-TOFMS analysis of sputum (Dao et al., 2021); Furthermore, Liu J et al. and Liu Q et al. precisely identified SNPT using some characteristic proteins (Liu et al., 2010; Liu et al., 2015).

To date, no studies have utilized proteomics to elucidate different molecular mechanisms in pathogenesis between SNPT and SPPT. In this study, we investigated proteome-level alterations in the plasma of SNPT, SPPT and control populations, aiming to identify specific protein signatures in the plasma of different TB patients and further elucidate the molecular mechanisms contributing to different disease pathogenesis.

## Materials and methods

### Participates

The blood samples were collected from 27 SPPT, 37 SNPT patients and 36 individuals without infection (Ctrl), and their demographic and clinical characteristics are shown in **Table S1**. The tuberculosis patients were recruited from the Tuberculosis Prevention and Treatment Institute of Kashgar, the Second People’s Hospital of Aksu, and the Kuqa County Infectious Disease Hospital. The Ctrl group were recruited from the First Affiliated Hospital of Xinjiang Medical University. Here, the inclusion criteria of TB patients included: 1) the people were diagnosed with tuberculosis based on clinical symptoms and microbiological evidence according to Diagnosis for Pulmonary Tuberculosis (WS 288-2017); 2) the patients provided the signed permissions for the use of their clinical data for scientific purpose and informed consent for the anonymous publication of data. The exclusion criteria included: 1) the TB patients in treatment period; 2) the TB patients with other chronic or acute diseases such as pregnancy complications, cardiac dysfunction, renal disease, psychiatric disease, gastrointestinal disease, uncontrolled hypertension, and some severe stress states (including cardiovascular and cerebrovascular events, severe infection, traumatic surgery, and severe wasting diseases). The control group were included according to the following criteria: no prior TB exposure, no specific clinical manifestations of TB, negative results on chest imaging, sputum smear test, and T-SPOT tests (Hu et al., 2022).

### Sample preparation

EDTA blood samples were collected from each participant and then centrifuged at 1500 g for 10 minutes at room temperature. The plasma was aliquoted and stored at -80°C. For protein preparation, high-abundance proteins were removed using the Agilent Human 14 Multiple Affinity Removal System according to the manufacturer’s instructions. Sample lysis and protein extraction were performed using SDT buffer (4% SDS, 100mM Tris-HCl, 1mM DTT, pH 7.6). The proteins were then digested overnight at 37°C with trypsin (Pierce, Thermo Fisher Scientific). The obtained tryptic peptides were desalted using C18 cartridges (Empore™ SPE Cartridges C18 (standard density), bed I.D. 7 mm, volume 3 mL, Sigma) and concentrated by vacuum centrifugation. Protein quantification was performed using the BCA Protein Assay Kit (Bio-Rad, USA). Protein integrity was confirmed by SDS-PAGE and silver staining, and samples showing protein degradation were excluded from the proteomic analysis. For metabolite preparation, the plasma samples were thawed on ice, and 150 μL of each sample was transferred to a centrifuge tube. Then, 1 mL of a chloroform-methanol solution was added, followed by ultrasound treatment. The supernatant was collected, and 2 mL of a 1% sulfuric acid-methanol solution was added. The mixture was methylated at 80°C for 30 minutes. Subsequently, 1 mL of n-hexane was added for extraction, and 5 mL of water was used for washing. Before injection, 500 μL of the extracted supernatant was mixed with 25 μL of an internal standard. This study was approved by the Hainan Province Clinical Medical Center.

### TMT-labeled quantitative proteomic analyses

The proteomic analyses were conducted by Shanghai Applied protein technology Co., Ltd (Shanghai, China) as previously reported protocol (Wei et al., 2018). The TMT 10plex labeling procedures (Thermo Fisher Scientific, San Jose, CA, US) were performed using the manufacturer’s instructions. Within each TMT experiment, three Ctrl samples were labeled with channels 126, 127N, and 127C. Three SNPT samples were labeled with channels 128N, 128C, and 129N. Three SPPT samples were labeled with channels 129C, 130N, and 130C. Additionally, a standard sample was created by mixing equal amounts of proteins from the nine samples and labeled with channel 131. For the labeling, each individual sample (100 μg) was resuspended in 100 μL of 0.1 M TEAB buffer, followed by reduction, alkylation, and trypsin digestion at 37°C for 16 hours. The reagents were dissolved in LC-grade acetonitrile (41 μL per 0.8 mg of reagent). After labeling for 1 hour, the reaction was quenched with 8 μL of 5% hydroxylamine for 15 minutes at room temperature. All channels were then mixed, aliquoted, and dried using a speed-vac. Subsequently, the TMT-labeled peptides were fractionated via high pH RP chromatography using a Zorbax 300 Extend-C18 column (3.5 μm, 4.6 × 250 mm; Agilent). LC-MS/MS analysis was performed on a Q Exactive mass spectrometer (Thermo Scientific) that was coupled to Easy nLC (Proxeon Biosystems, now Thermo Fisher Scientific) for 60/90 min.

The peptide mixture was loaded onto a reverse phase trap column (Thermo Scientific Acclaim PepMap100, 100 μm*2 cm, nanoViper C18) connected to the C18-reversed phase analytical column (Thermo scientific EASY column, 10 cm, ID75 μm, 3 μm, C18-A2) in buffer A (0.1% formic acid), and then separated with a linear gradient of buffer B (84% acetonitrile and 0.1% Formic acid) at a flow rate of 300 nL/min by Intelli Flow technology. LC–MS/MS analysis was operated in positive ion mode. Full MS scans were acquired in the mass range of 300-1800 m/z. Automatic gain control (AGC) target was set to 1e6, and maximum inject time to 50 ms. The dynamic exclusion duration was set for 60.0 s. Survey scans were acquired at a resolution of 70,000 at m/z 200 and resolution for HCD spectra was set to 35,000 at m/z 200, and isolation width was 2 m/z. Normalized collision energy was 30 eV and the underfill ratio was defined as 0.1%. Proteome Discoverer (v.1.4) was used to search all the Q Exactive raw data. False discovery rate (FDR) was set as ≤0.01. The protein ratios were calculated as the median of only unique peptides of the protein. All peptide ratios were normalized by the median protein ratio, and the median protein ratio should be “1” after the normalization.

### Targeted protein quantification by LC-PRM/MS

LC-PRM/MS analysis were performed on 32 DEPs obtained from the TMT quantitative proteomic analysis (Shanghai Applied Protein Technology Co., Ltd, China) using the procedure described previously (Cao et al., 2022). Briefly, peptides were first prepared, with an internal standard reference of a Peptide Retention Time Calibration Mixture (Thermo Scientific). Tryptic peptides were desalted using C18 stage tips and subjected to reversed-phase chromatography on an Easy nLC-1200 system (Thermo Scientific) with 1-hour gradients. PRM analysis was performed on a Q Exactive HF mass spectrometer (Thermo Scientific) using optimized parameters for collision energy, charge state, and retention times. The mass spectrometer was operated in positive ion mode with specific parameters: full MS1 scan resolution of 70,000, AGC target values of 3.0×10^-6^, and a maximum ion injection time of 200 ms. This was followed by 20 PRM scans at 35,000 resolution (at m/z 200) with AGC set to 3.0×10^-6^ and a maximum injection time of 200 ms. Targeted peptides were isolated with a 1.5 Th window, and ion activation/dissociation was performed at a normalized collision energy of 27 using higher energy dissociation (HCD) in the collision cell. Data analysis was conducted using Skyline (MacCoss Lab, University of Washington), quantifying signal intensities for significantly altered proteins relative to each sample with normalization to a standard reference.

### Targeted lipidomics by GC-MS analysis

The metabolic properties of fatty acids obtained from proteomic analyses were then measured by targeted lipidomics using GC-MS analysis as previously described (Wen et al., 2020). In brief, the sample of metabolites were separated on an Agilent DB-WAX capillary column (30 m × 0.25 mm ID × 0.25 μm) gas chromatography system. The temperature programming was as follows: 50°C for 3 min, 220°C for 20 min, and 250°C for 10 min. An Agilent 6890N/5975B gas chromatography-mass spectrometer was used for analysis. The electron bombardment ionization (EI) source, SIM scanning mode, and electron energy were 70 eV.

### Functional annotation and statistical analysis of differentially expressed proteins

We analyzed the differentially expressed proteins (|FC| ≥ 1.2, P-value < 0.05) performing pathway and functional annotation analysis using the Ingenuity Pathway Analysis (IPA, QIAGEN, USA) platform. We conducted core and comparison analyses to obtain canonical pathway and disease and biofunctions, which were evaluated by P-value (P-value < 0.05) and Z-score (Z-score ≠ 0). P-value < 0.05 indicated that the item was significantly enriched; Z-score > 0 indicated that the item was activated and Z-score < 0 indicated that the item was inhibited. Statistical analyses were performed using R software v4.1.0, and P-value < 0.05 indicated statistical significance.

## Results

### Altered protein expression profiles in SNPT and SPPT

**Figure 1:**
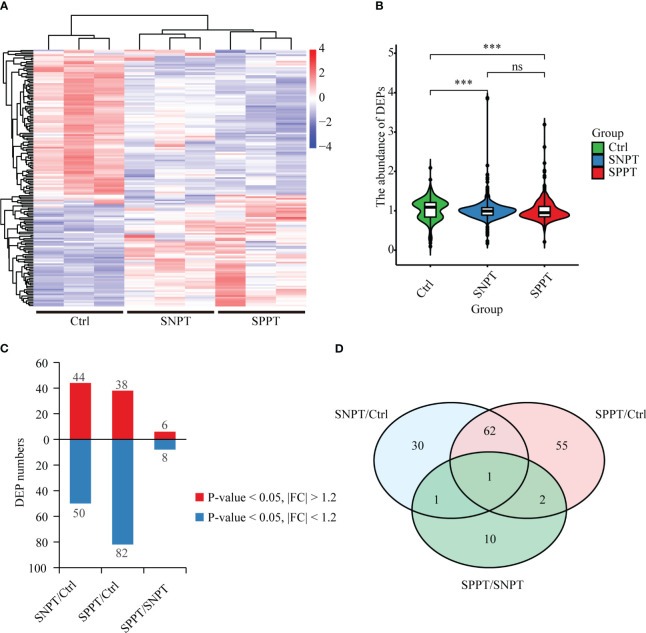
Protein expression profiles among SNPT, SPPT and Ctrl samples. **(A)** Heatmap showing protein expression profiles among the three groups. **(B)** Bar plot displaying the up-regulated and down-regulated DEPs in the SNPT/Ctrl, SPPT/Ctrl and SPPT/SNPT groups. **(C)** Violin plot showing the abundance of DEPs with downward trend from Ctrl to SNPT and then to SPPT. **(D)** Venn diagram showing the overlap among the three groups. ***p<0.001.

To explore the molecular mechanisms of SPPT and SNPT patients, we performed TMT quantitative proteomic analyses on plasma samples from 27 SPPT, 37 SNPT patients, and 36 Ctrl individuals (**Table S1**). The results displayed distinct protein expression profiles among the three groups, and 161 differentially expressed proteins (DEPs) were identified (|FC| > 1.2, P-value < 0.05) (**Figures 1A, B**; **Table S2: Additional Table 1**). Compared with Ctrl, 44 up-regulated and 50 down-regulated DEPs were obtained in SNPT, and 38 up-regulated and 82 down-regulated DEPs were detected in SPPT (**Figure 1C**); 30 and 55 DEPs were specially expressed in the SNPT/Ctrl and SPPT/Ctrl groups, respectively (**Figure 1D**). Some of them were further verified by PRM targeted proteomic mass spectrometry (**Figure S1**), which displayed a consistent pattern obtained from TMT analysis.

### Expression panels and patterns of plasma proteins showing increased immune response and lipid metabolic disorder in SNPT and SPPT patients

**Figure 2:**
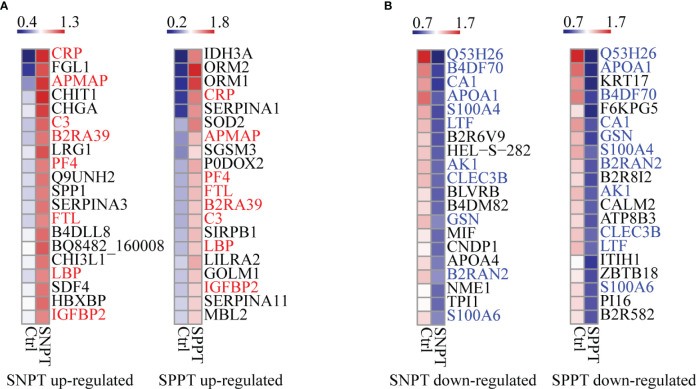
Heatmaps showing the top-20 upregulated and downregulated DEPs in the SNPT and SPPT samples. **(A)** The top-20 up-regulated DEPs in the SNPT and SPPT samples. Among them, eight proteins (labeled with red color) are shared in both SNPT and SPPT. **(B)** The top-20 down-regulated DEPs in SNPT and SPPT samples. Among them, 11 proteins (labeled with blue color) are shared in both SNPT and SPPT. The color key indicates the protein expression levels that range from blue (low expression) to red (high expression).

We then analyzed the top 20 up- and down-regulated DEPs in the SNPT/Ctrl and SPPT/Ctrl groups (**Figure 2**). Of the top 20 up- and down-regulated DEPs, eight and 11 were shared by the two groups (**Figures 2A, B**).

For the top 20 up-regulated DEPs (**Figure 2A**; **Table S3**), seven of eight shared DEPs were associated with inflammation/immune response (CRP, APMAP, C3, B2RA39, PF4, LBP and IGFBP2), indicating increased inflammatory response in TB patients regardless of SNPT or SPPT. Further analysis about 12 specially up-regulated DEPs showed the stronger immune response in SPPT than in SNPT: seven of the 12 specifically upregulated DEPs in the SPPT/Ctrl group were associated with innate immune response (ORM2, ORM1, P0DOX2, SIRPB1, LILRA2, GOLM1, and MBL2), while five of the specifically upregulated DEPs in the SNPT/Ctrl group were related to anti-inflammation (FGL1, CHIT1, LRG1, SERPINA3, and B4DLL8).

For the top-20 down-regulated DEPs (**Figure 2B**; **Table S3**), nine of 11 shared DEPs in the two groups were involved in metabolism and antioxidant stress (Q53H26, B4DF70, CA1, APOA1, S100A4, LTF, AK1, B2RAN2 and S100A6), demonstrating decreased metabolism and antioxidant capacity in TB patients. On the other hand, in the SNPT/Ctrl group, seven out of nine DEPs that were specifically down-regulated were also related to metabolism and antioxidant stress (HEL-S-282, BLVRB, B4DM82, CNDP1, APOA4, NME1, and TPI1). This observation suggested a decrease in metabolism and antioxidant capacity in SNPT patients. Among the nine specifically down-regulated DEPs in the SPPT/Ctrl group, four were related to lipid metabolism (KRT17, B2R8I2, ATP8B3, and PI16), and the remaining five were associated with injury/remodeling processes (F6KPG5, CALM2, ITIH1, ZBTB18, and B2R582). These findings indicated an inhibition of lipid metabolism and damage repair processes in SPPT patients.

Additionally, we detected six up-regulated and eight down-regulated DEPs in the SPPT/SNPT group. For the up-regulated DEPs, five were associated with inflammation/immune responses; for the down-regulated DEPs, five were related to metabolism, two of which were associated with lipid metabolism (PGLYRP2 and KNG1) (**Table S4**). These reflected disordered immune responses and metabolism compared to SNPT.

**Figure 3:**
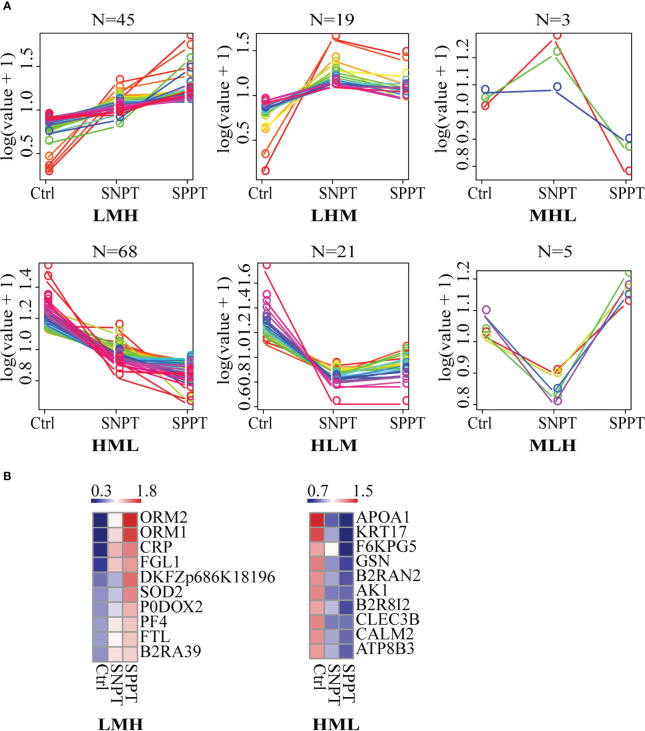
The expression patterns of DEPs among the Ctrl, SNPT, and SPPT samples. **(A)** Six protein expression patterns (including LMH, LHM, MHL, HML, MLH, and HLM). L, low expression level; M, medium expression level; H, high expression level; N, the protein numbers of each pattern. **(B)** Heatmaps showing the top 10 continuously upregulated/downregulated DEPs from Ctrl to SNPT and then to SPPT (LMH and HML). Colors indicate the protein expression levels that range from blue (low expression) to red (high expression).

We then classified all DEPs into six expression patterns based on the protein expression trends among the three groups (**Figure 3A**; **Table S5, S6**), and further focused on the top10 proteins in the LMH and HML patterns with continuously up- or down-regulated expression trends from Ctrl to SNPT and then to SPPT (**Figure 3B**). Of the top10 proteins in the LMH pattern, six were related to innate immune regulation (ORM2, ORM1, CRP, FGL1, P0DOX2, and PF4), indicating continuously enhanced body inflammatory response as increasing bacterial loads. Conversely, among the top 10 proteins of HML pattern, four were related to lipid metabolism, including APOA1, KRT17, B2R8I2, and ATP8B3, demonstrating continuously increased lipid metabolism disorder with the increase of bacterial loads.

For the LHM and MHL patterns (with higher expressed proteins in SNPT compared to SPPT) (**Figure S2**; **Table S6**), six out of 13 proteins (~46%), were related to anti-inflammatory responses, indicating their crucial role in protecting the body from excessive inflammatory responses in SNPT. For the 15 DEPs in the HLM and MLH patterns (with lower expressed proteins in SNPT compared to SPPT), four were associated with immune response (B2R6V9, DDT, B2RBZ5, and S6B294), and 10 were related to metabolism and antioxidant stress (**Figure S2**; **Table S6**).

### IPA analysis revealing dysfunctional immune response and metabolism in the SNPT and SPPT patients

To investigate the molecular mechanism of SPPT and SNPT, we further performed pathway and functional analyses using IPA. First, 40 significantly enriched pathways (P-value < 0.05) were identified in the SNPT/Ctrl and SPPT/Ctrl groups (**Table S7, Table S2: Additional Table 2** and **Additional Table 4**). Among the top 10 shared significantly enriched pathways in the two groups (**Figure S3**), five were related to immune response (“Acute Phase Response Signaling”, “Production of Nitric Oxide and Reactive Oxygen Species in Macrophages”, “IL-12 Signaling and Production in Macrophages”, “Clathrin-mediated Endocytosis Signaling”, and “Granulocyte Adhesion and Diapedesis”), and three were associated with lipid metabolism (“LXR/RXR Activation”, “FXR/RXR Activation”, and “Atherosclerosis Signaling”). These indicated dysfunctional immune response and lipid metabolism in the SNPT and SPPT patients.

**Figure 4:**
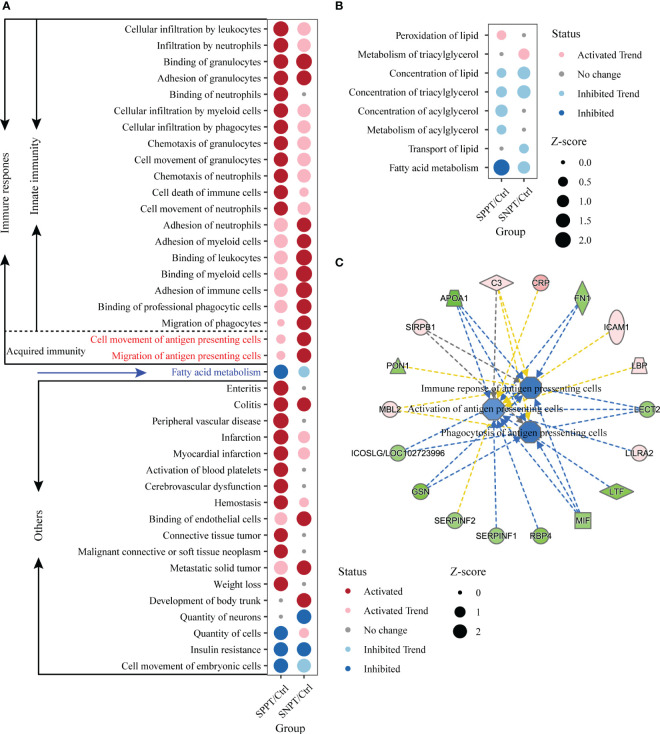
IPA function analyses of immune response in the SNPT/Ctrl and SPPT/Ctrl groups. **(A)** Dumbbell chart showing the significantly activated/inhibited functions in two groups. **(B)** Dumbbell chart showing the enriched lipid metabolism related functions in the two groups. The dark red represents significantly upregulated functions (p-value < 0.05, Z-score ≥ 2); the light red represents upregulated functions (p-value < 0.05, 0 < Z-score < 2). The dark blue represents significantly downregulated functions (p-value < 0.05, Z-score ≤ -2); the light blue represents downregulated functions (p-value < 0.05, -2 < Z-score < 0). **(C)** Networks showing the enriched DEPs in the antigen presenting cell related functions in SPPT/Ctrl group. Light red: increased measurement; green: decreased measurement; orange: predicted activation; blue: predicted inhibition; yellow: findings inconsistent with state of downstream molecule; solid line: direct interaction; dashed line: indirect interaction.

500 significantly enriched biofunctions (P-value < 0.05) were then identified in the SNPT/Ctrl group and SPPT/Ctrl groups (**Table S8**, **Table S2**: **Additional Table 3** and **Additional Table 5**), among which 40 were significantly activated/inhibited in at least one group (|Z-score| ≥ 2, **Figure 4A**). Herein, 21 of the 40 functions (more than half) belonged to immune related functions (**Figure 4A**), all of which were significantly activated (Z-score ≥ 2) in at least one group; fatty acid metabolism was significantly inhibited in the SPPT/Ctrl group. IPA biofunction analysis also showed dysfunctional immune and fatty acid metabolism in SNPT and SPPT.

### Gradual inhibition of fatty acid metabolism from Ctrl to SNPT then to SPPT

We further explored fatty acid metabolism in SPPT and SNPT patients, and found gradually inhibition of fatty acid metabolism from Ctrl to SNPT then to SPPT (**Figure 4A**). Among eight enriched lipid metabolism related functions in the two groups (P-value < 0.05), it was worth noting that only one lipid metabolism related function (“Fatty acid metabolism”) was significantly inhibited (Z = -2.177) in the SPPT/Ctrl group, while it was inhibited (Z = -1.052) in the SNPT/Ctrl group (**Figure 4B**). This indicated more serious inhibition of fatty acid metabolism in SPPT than in SNPT, as previously reported in our previous metabolic findings about SPPT and SNPT (Hu et al., 2022).

To verify the above findings, we further performed targeted lipidomics using GC-MS analysis (Wen et al., 2020). The results indeed showed more severe inhibition of lipid metabolism in SPPT than in SNPT, especially for unsaturated fatty acids (**Figure S4**, **Table S2**: **Additional Table 6**). In the SPPT/Ctrl group, eight out of 17 fatty acid molecules showed downregulation, among which six displayed significant downregulation with P-value < 0.05, including two polyunsaturated fatty acids (PUFA); in the SNPT/Ctrl group, most fatty acid molecules (14/17 = 82.4%) showed up-regulation, among which nine displayed significant upregulation with P-value < 0.05, including six unsaturated fatty acid molecules.

### Increased innate immune response but decreased movement of antigen-presenting cells in SPPT patients compared to SNPT patients

To explore the immune mechanism of SPPT and SNPT, we focused on 21 immune-related biofunctions, containing 19 innate- and two acquired-immune related ones (**Figure 4A**). In the SNPT/Ctrl group, all 11 immune-related biofunctions (including two shared and nine specifically enriched ones) were associated with migration/movement and binding/adhesion of immune cells, among which two specifically enriched ones belonged to acquired immune-related biofunctions and were related to antigen-presenting cell movement. In the SPPT/Ctrl group, five of 12 immune-related functions (including two shared ones) were associated with migration/movement and binding/adhesion of immune cells. The other ten specifically enriched items were related to movement, chemotaxis, infiltration and even death of immune cells, suggesting stronger innate immune response in SPPT patients.

Notably, in the SNPT/Ctrl group, two acquired immune-related biofunctions, “Cell movement of antigen presenting cells” (P-value < 0.05, Z-score = 2.084) and “Migration of antigen presenting cells” (P-value < 0.05, Z-score = 2.179) were significantly activated (**Figure 4A**), while they were not significantly activated in the SPPT/Ctrl group (Z-score = 0.576 and Z-score = 0.478). In addition, we also detected three down-regulated antigen presenting cells related functions in the SPPT/Ctrl group [(“Activation of antigen presenting cells” (Z-score = -1.028); “Phagocytosis of antigen presenting cells”, Z-score = -1.577; “Immune response of antigen presenting cells”, Z-score = -1.482)] (**Figure 4C**). A significantly down-regulated inducible T cell co-stimulator ligand/key molecule of activating T cells (ICOSLG/LOC102723996) (P-value < 0.05, FC = 0.82) were also detected in SPPT patients. The above findings suggested that antigen-presenting cell activation might be a critical determinant of different bacterial loads/symptoms between SNPT and SPPT.

### Stronger activation of antigen presenting/acquired immune related functions/proteins in SNPT than in SPPT

**Figure 5:**
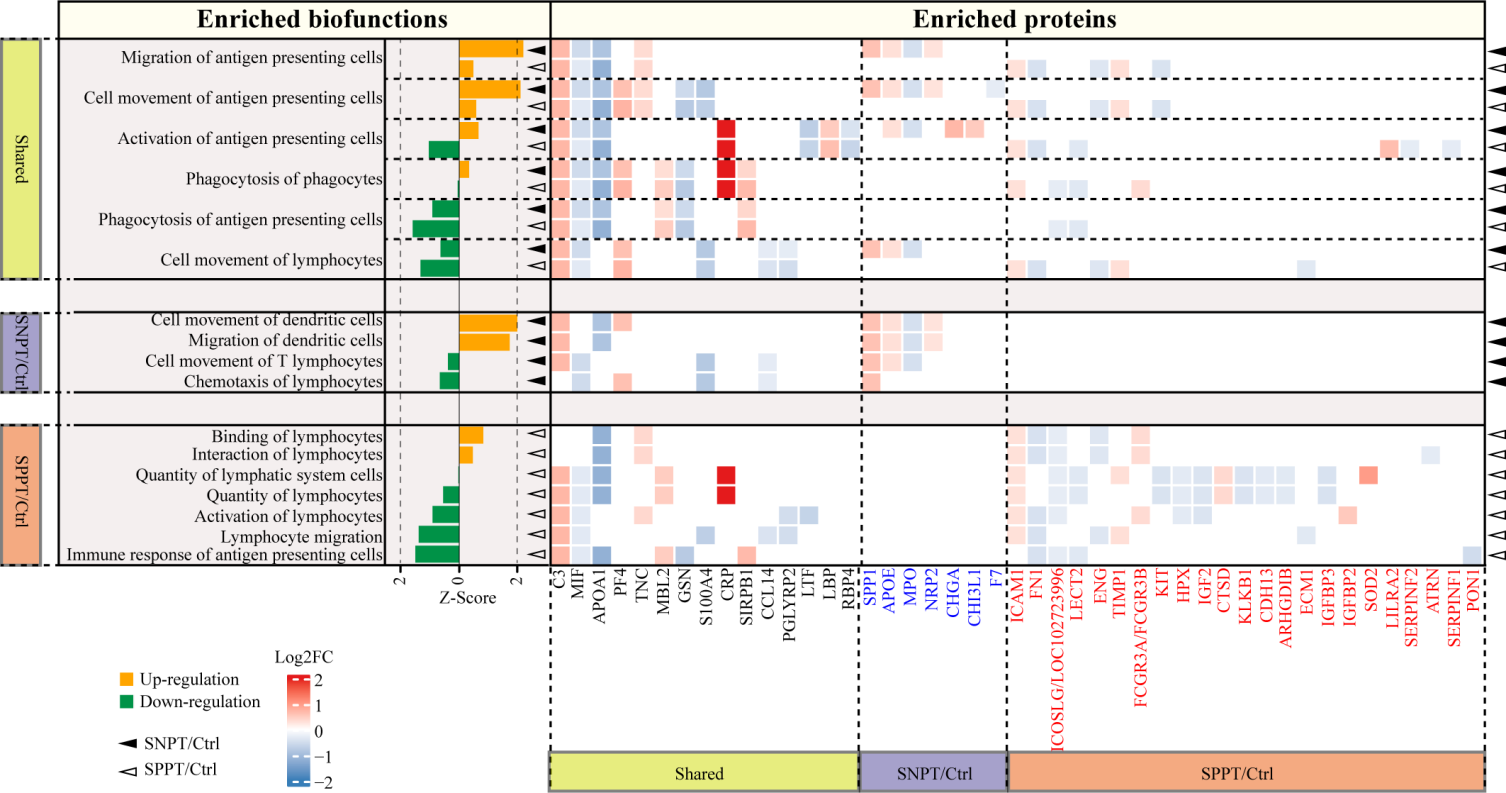
Acquired immune-related functions and the corresponding DEPs in the SNPT/Ctrl and SPPT/Ctrl groups. Bar plot showing the acquired immune-related functions in the two groups. Orange and green bars indicate the up- (Z-score > 0, P-value < 0.05) and down-regulation (Z-score < 0, P-value < 0.05), respectively. The heatmap depicts the DEPs and their expression level in each biofunction item. DEPs highlighted in blue and red font correspond to proteins specifically enriched in SNPT/Ctrl and SPPT/Ctrl groups, respectively. The solid triangles and hollow triangles in each row represent the enriched functions and their corresponding DEPs in the SNPT/Ctrl and SPPT/Ctrl groups, respectively. Left and bottom labels: “Shared” indicates the functions and DEPs shared by the SNPT/Ctrl and SPPT/Ctrl groups; “SNPT/Ctrl” and “SPPT/Ctrl” represent the functions and DEPs that are specific to the SNPT/Ctrl and SPPT/Ctrl groups, respectively.

To further investigate antigen-presenting and acquired immunity in SNPT and SPPT, we analyzed all enriched acquired immune-related functions and corresponding DEPs (**Figure 5**; **Table S9**). The results showed stronger activation of antigen presenting and acquired immune related functions/proteins in SNPT than in SPPT. Herein, a total of 17 functions related to antigen-presenting and acquired immunity were identified in the two groups, including six shared ones, four specifically enriched ones in SNPT, and seven specifically enriched ones in SPPT.

First, among the six shared functions, all of them showed stronger activation or weaker inhibition of antigen presenting and acquired immune in SNPT than in SPPT. For antigen-presenting, in addition to the two significantly activated ones in SNPT (described in the previous section), “Activation of antigen-presenting cells” function was activated in SNPT but inhibited in SPPT, and the inhibition degree of “Phagocytosis of antigen-presenting cells” function was lower in SNPT than in SPPT. Secondly, among the four significantly enriched functions in SNPT, half of them were related to antigen-presenting and activated. Thirdly, among the seven significantly enriched functions in SPPT, most of them were inhibited, including “Immune response of antigen-presenting cells”. Overall, compared to SNPT, the inhibition of antigen-presenting cells (APCs) activation in SPPT might cause impaired acquired immunity, subsequently resulting in a higher bacterial load in SPPT than in SNPT.

Further analysis revealed that some proteins might be associated with differential antigen-presenting and acquired immune responses between the two groups. First, a total of 45 DEPs were enriched in the aforementioned APC/acquired immune related functions. Among them, 15 were shared in the two groups and exhibited consistent expression trends in both SNPT and SPPT patients, which were therefore not subjected to further analysis. The remaining 30 proteins were specifically enriched in the acquired immunity-related functions of either SNPT or SPPT, suggesting that they might play crucial roles in differential genotypes and phenotypes between SPPT and SNPT.

For seven specifically enriched proteins in SNPT, four of five up-regulated proteins (SPP1, CHI3L1, APOE, and NRP2) have been reported to be involved in promoting T cell activation and facilitating pathogen clearance (van den Elzen et al., 2005; Dela Cruz et al., 2012; Shan et al., 2014; Roy et al., 2017); one of two down-regulated proteins (MPO) has been reported to be associated with inhibition of antigen presenting cell activation (Odobasic et al., 2013).

For 23 specifically enriched proteins in SPPT, six of seven up-regulated proteins (SOD2, LILRA2, IGFBP2, FCGR3A/FCGR3B, TIMP1, and ICAM1) were associated with suppression of antigen presentation and T-cell activation, and upregulation of Treg(Marchesi et al., 2013; O'Mahony et al., 2016; Seo et al., 2019; Keller et al., 2021; Robilliard et al., 2022; Zhang et al., 2022). Here, Treg cells have been reported to play roles in suppressing a variety of immune cell activation, including B cells, CD4+, and CD8+ T cells (Seo et al., 2019). On the other hand, nine of 16 down-regulated proteins (ICOSLG/LOC102723996, ATRN, CDH13, ECM1, ARHGDIB, LECT2, IGF2, FN1, and PON1) have been reported to play roles in antigen presenting and T cell activation(Tang et al., 2000; Chitnis and Khoury, 2003; Zhang et al., 2006; Jodaa Holm et al., 2016; Bai et al., 2018; Bijen et al., 2018; He et al., 2018; Luo et al., 2019; Belfiore et al., 2023).

## Discussion

Our proteomic and lipidomic study revealed differential immune and metabolic mechanisms/microenvironments underling different clinical phenotypes between SPPT and SNPT plasma samples. Although both groups displayed activated innate immune responses and inhibited fatty acid metabolism, SPPT patients showed stronger activation of innate immune responses and inhibition of fatty acid metabolism than SNPT patients. Importantly, our analyses on acquired immune-related functions uncovered activated APCs in SNPT but inhibited APCs in SPPT, which might be a critical determinant of different bacterial loads and phenotypes/symptoms between SNPT and SPPT. Specifically, the activation of APCs in tuberculosis has been reported to stimulate cellular/acquired immune responses and effectively eliminate the majority of *Mtb* strains in bodies (Cooper and Khader, 2008), leading to lower bacterial loads. Conversely, APC activation is inhibited in SPPT, resulting in inhibited acquired immune responses and further losing control of bacterial loads.

The foregoing conclusion were further validated by the analysis for related DEPs enriched in APC/acquired immune related functions (**Figure 5**; **Table S9**), also providing some promising therapeutic targets for TB. Among the specifically enriched proteins in SNPT, four specifically up-regulated proteins in SNPT have been reported to be involved in promoting T cell activation and facilitating pathogen clearance (van den Elzen et al., 2005; Dela Cruz et al., 2012; Shan et al., 2014; Roy et al., 2017). SPP1 is required for mDC-dependent activation of T cell subsets (Shan et al., 2014); CHI3L1 facilitates killing bacteria in macrophage by inhibiting caspase-1-dependent macrophage pyroptosis (Dela Cruz et al., 2012); APOE and lipid antigens can be delivered into endosomal compartments of APCs together to activate T-cells activation (van den Elzen et al., 2005); NRP2 is required for T cell activation through DCs migration in response to CCL21 (Roy et al., 2017). On the other hand, one specifically down-regulated protein MPO in SNPT has been reported to inhibit DC activation and antigen uptake/processing (Odobasic et al., 2013).

Among the specifically enriched proteins in SPPT, six up-regulated proteins have been reported to be associated with suppression of antigen presentation and T-cell activation, and upregulation of Treg (Marchesi et al., 2013; O'Mahony et al., 2016; Seo et al., 2019; Keller et al., 2021; Robilliard et al., 2022; Zhang et al., 2022). Overexpression of SOD2 facilitates the expression of FoxP3 (a major transcription factor of Treg cells) (Seo et al., 2019); LILRA2 is an immune modulator by inhibiting DC differentiation or antigen presenting capacity by stimulating an alternative macrophage differentiation pathway (Marchesi et al., 2013); IGFBP2 suppresses the influx of T-cells (Robilliard et al., 2022); FCGR3A (CD16a) plays a role in immature lanDCs, plasmacytoid DCs, or CD1c+ DCs O'Mahony et al., 2016); TIMP1 overexpression leads to Treg upregulation (Keller et al., 2021); ICAM-1-mediated adhesion is a prerequisite for exosome-induced T cell suppression (Zhang et al., 2022).

On the other hand, nine specifically downregulated proteins in SPPT have been reported to play roles in APC and T cell activation (Tang et al., 2000; Chitnis and Khoury, 2003; Zhang et al., 2006; Esensten et al., 2016; Jodaa Holm et al., 2016; Bai et al., 2018; Bijen et al., 2018; He et al., 2018; Luo et al., 2019; Belfiore et al., 2023). ICOSLG/LOC102723996 (an inducible T cell costimulatory molecule) is required for the second signal of T cell activation(Chitnis and Khoury, 2003; Esensten et al., 2016); ATRN can be released by activated T lymphocytes (Tang et al., 2000); CDH13 -derived peptide can activate T cell clone (Bijen et al., 2018); ECM1 is a positive regulator of Tfh differentiation and antibody production (He et al., 2018); ARHGDIB facilitates the connection between antigen-presenting cells and T cells by promoting T cell proliferation (Zhang et al., 2006); LECT2 plays roles in Th1 and Th17-guided immune responses (Jodaa Holm et al., 2016); IGF2 induces dendritic cell maturation (Belfiore et al., 2023); FN1 enhances T cell response in lung cancer cells (Luo et al., 2019); it is worth noting that PON1 is necessary for thymocyte development from double-negative to double-positive transition stages (Bai et al., 2018), therefore its significant downregulation in SPPT patients can inhibit T cell activation (Bai et al., 2018).

Collectively, four specifically upregulated and one specifically downregulated protein in SNPT have been reported to enhance antigen-presenting cell activation and cellular immunity/acquired immune responses, further resulting in more effective clearance of *Mtb* strains in bodies. On the other hand, these SPPT-specific enriched proteins (six upregulated and nine downregulated ones) are able to suppress antigen-presenting cell and cellular immunity in SPPT patients, leading to a higher bacterial load in bodies.

In addition, our expressed pattern analysis revealed differential immune responses between SNPT and SPPT (**Figure S2**; **Table S6**). Specifically, six out of the 13 higher expressed proteins in SNPT are related to anti-inflammatory response, indicating weaker immune response and delicate balance of immune responses in SNPT (**Table S6**): four (CHIT1, LRG1, B7Z4R8, and B4DLL8) have been reported to be involved in macrophage M2 polarization; two (SERPINA1 and B7Z7M2) are identified as acute-phase reactive proteins for protecting the body from excessive inflammatory responses. On the other hand, four out of 15 higher expressed proteins in SPPT (B2R6V9, DDT, B2RBZ5, and S6B294) are associated to immune responses, suggesting stronger immune response in SPPT.

Our expressed pattern and panel analyses also revealed differential lipid metabolism between SNPT and SPPT (**Figure 3**; **Table S5**). Notably, four lipid related proteins showed a continuously down-regulation trend from Ctrl to SNPT and then to SPPT (APOA1, KRT17, B2R8I2, and ATP8B3), reflecting gradual inhibition of lipid metabolism from SNPT to SPPT (**Table S5**): APOA1 can facilitate the excretion of cholesterol and phospholipids in cell; KRT17 has been proved to help to lipid metabolism-related enzyme and antimicrobial peptide expressions; B2R8I2 and ATP8B1 are identified to involve in bile acid and lipid metabolism. In addition, two down-regulated DEPs (PGLYRP2 and KNG1) in the SPPT/SNPT group are associated with lipid metabolism, indicating more dysfunctional lipid metabolism in SPPT (**Table S4**).

Nevertheless, we have observed consistent expression trends of some shared upregulated/downregulated functions or proteins in both SNPT and SPPT (**Figures 3**–**5**), revealing phenotype generality of TB (both SNPT and SPPT). For SNPT patients, there is a delicate balance between immune responses and bacterial loads: on the one hand, some activated innate and acquired immune responses (especially APC activation) are able to clear up the majority of *Mtb* strains in bodies; on the other hand, some immunosuppression-related functions/proteins restrict complete *Mtb* eradication. For SPPT, since only some innate immune responses are activated while the acquired immune response may be inhibited leading to higher bacterial loads due to broken balance.

Overall, our findings reveal differential immune and metabolic mechanisms/microenvironments underling SPPT and SNPT patients, and highlight the critical role of antigen-presenting cells in SNPT for clearing up the majority of *Mtb* in bodies. This suggests the important role of antigen-presenting cell in promoting acquired immunity for inhibiting *Mtb* infection. Some proteins are further verified to play important roles in the APC activation/acquired immune response process, providing some promising therapeutic promising therapeutic targets for TB. Further studies on these are warranted.

Although our study provides important insights into the pivotal role of APCs in SNPT patients in clearing the infection, it is important to validate the result by experiments. Regrettably, we are unable to conduct such validation experiments due to the revocation of our license to handle *Mtb* infections in animals, a decision made by the Xinjiang government impacting our institution, the First Affiliated Hospital of Xinjiang Medical University. Nonetheless, our findings shed light on the significance of APC-mediated immune responses in countering *Mtb* infections. Further studies on these are warranted by animal experiments.

## Data availability statement

The datasets presented in this study can be found in online repositories. The names of the repository/repositories and accession number(s) can be found below: https://ngdc.cncb.ac.cn/omix/, OMIX004336.

## Ethics statement

This study was approved by the Ethical Committee of First Affiliated Hospital of Xinjiang Medical University (Record number 20171123-06-1908A) and project supported by Hainan Province Clinical Medical Center. All enrolled subjects provided written informed consent. All methods were performed in accordance with the relevant guidelines and regulations. The studies were conducted in accordance with the local legislation and institutional requirements. The participants provided their written informed consent to participate in this study.

## Author contributions

FC and JingW conceived the study. YJ, JieW, and CL performed the bioinformatics analyses. CJ, SC, BT, YL, YS, and WQ carried out the experimental analyses. YJ, JieW, CL, XW, PW, LY, and XJ drew the figures. FC and YJ wrote the manuscript. All authors read and approved the final manuscript.

## Supplementary material

Supplementary Figure 1 Verification of 15 proteins using LC-PRM/MS. Fifteen out of 32 selected proteins and verified using PRM targeted proteome mass spectrometry. The expression trends of these proteins were found to be consistent with the TMT results. The colors in the figure represent the expression levels of differentially expressed proteins, ranging from blue (low expression) to red (high expression).

Supplementary Figure 2 Heatmaps showing the DEPs in the four patterns (LHM, MHL, HLM and MLH). Colors indicate the protein expression levels that range from blue (low expression) to red (high expression).

Supplementary Figure 3 The top 10 significantly enriched items (P-value < 0.05) in both SNPT/Ctrl and SPPT/Ctrl groups. Here most pathways are related to immune response and metabolism (labeled with red and blue, respectively).

Supplementary Figure 4 Bar plot showing the abundance change of 17 fatty acid molecules in the SNPT/Ctrl and SPPT/Ctrl groups. Red and blue bars indicate up- and down-regulation, respectively. The left and right panels represent the SNPT/Ctrl and SPPT/Ctrl groups, respectively.
