# Supplementary figures and images for: Proteomic analyses of smear-positive/negative tuberculosis patients uncover differential antigen-presenting cell activation and lipid metabolism
Source: Front Cell Infect Microbiol. 2023 Oct 16;13:1240516. doi: 10.3389/fcimb.2023.1240516 (PMC10613889; doi:10.3389/fcimb.2023.1240516)

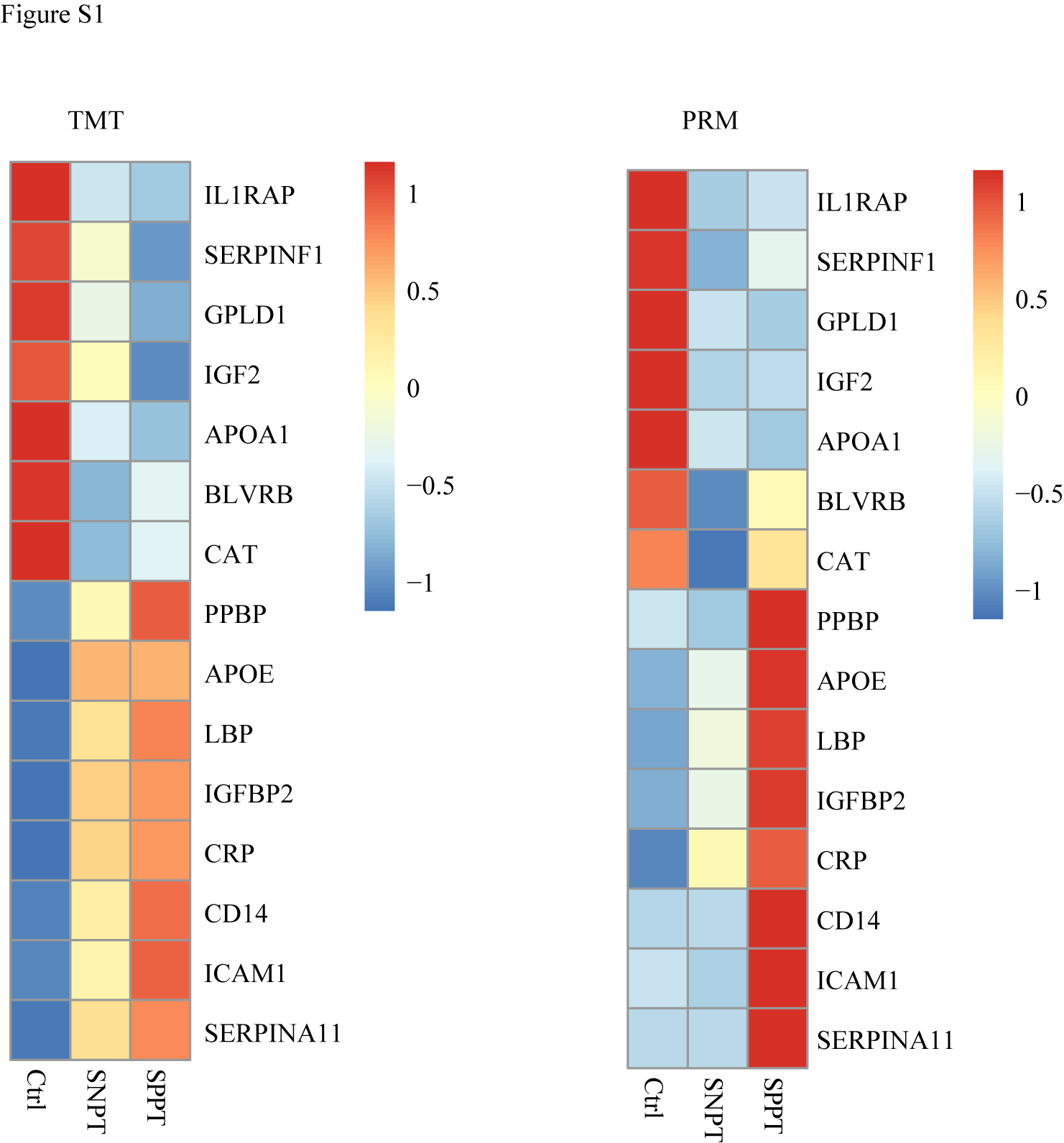

Supplement: Supplementary Figure 1 — Verification of 15 proteins using LC-PRM/MS. Fifteen out of 32 selected proteins and verified using PRM targeted proteome mass spectrometry. The expression trends of these proteins were found to be consistent with the TMT results. The colors in the figure represent the expression levels of differentially expressed proteins, ranging from blue (low expression) to red (high expression). [file Image_1.tif]

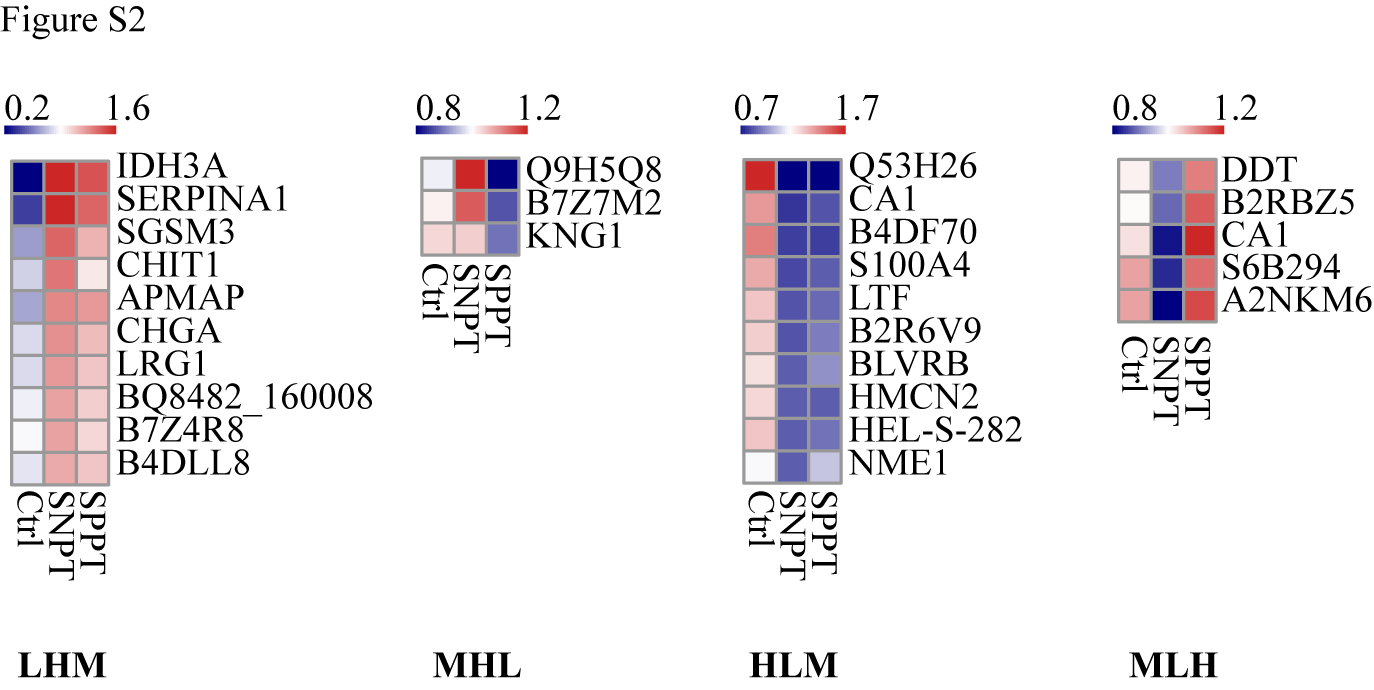

Supplement: Supplementary Figure 2 — Heatmaps showing the DEPs in the four patterns (LHM, MHL, HLM and MLH). Colors indicate the protein expression levels that range from blue (low expression) to red (high expression). [file Image_2.tif]

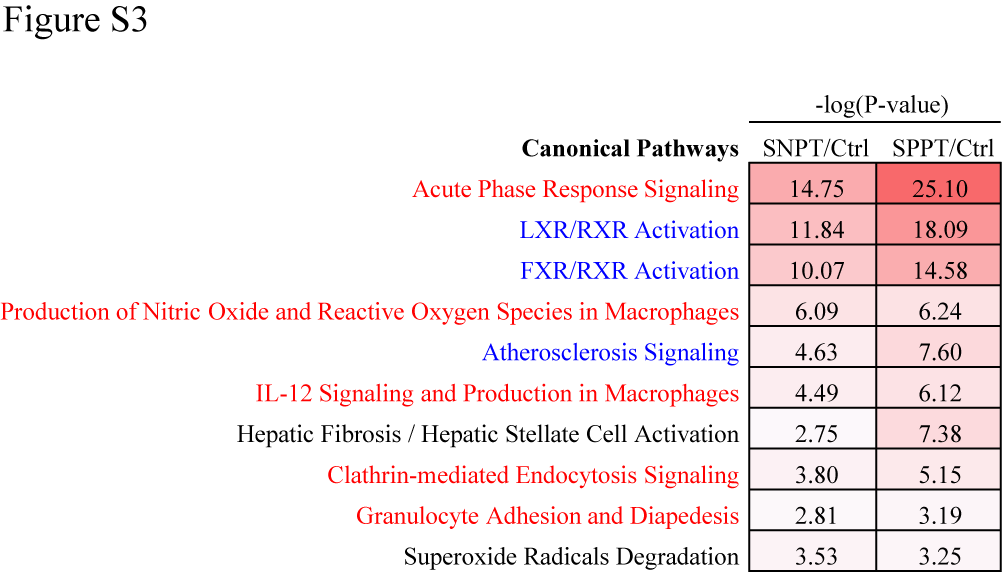

Supplement: Supplementary Figure 3 — The top 10 significantly enriched items (P-value < 0.05) in both SNPT/Ctrl and SPPT/Ctrl groups. Here most pathways are related to immune response and metabolism (labeled with red and blue, respectively). [file Image_3.tif]

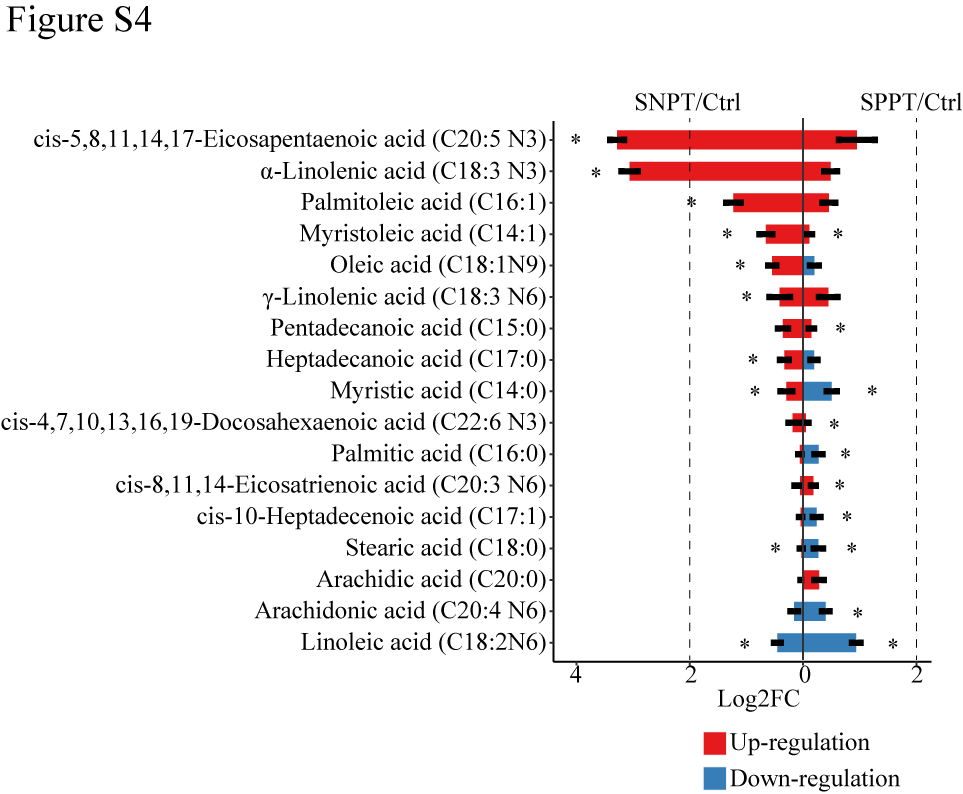

Supplement: Supplementary Figure 4 — Bar plot showing the abundance change of 17 fatty acid molecules in the SNPT/Ctrl and SPPT/Ctrl groups. Red and blue bars indicate up- and down-regulation, respectively. The left and right panels represent the SNPT/Ctrl and SPPT/Ctrl groups, respectively. [file Image_4.tif]
